# 2-[(2-Meth­oxy­benzyl­idene)amino]­phenol

**DOI:** 10.1107/S1600536812016224

**Published:** 2012-04-21

**Authors:** M. Aslam, I. Anis, N. Afza, M. T. Hussain, S. Yousuf

**Affiliations:** a Pharmaceutical Research Centre, PCSIR Laboratories Complex, Karachi, Pakistan and Department of Chemistry, University of Karachi, Karachi, Pakistan; b Department of Chemistry, University of Karachi, Karachi, Pakistan; c Department of Applied Sciences, National Textile University, Faisalabad 37610, Pakistan; d H.E.J. Research Institute of Chemistry, International Center for Chemical and Biological Sciences, University of Karachi, Karachi 75270, Pakistan

## Abstract

In the title compound, C_14_H_13_NO_2_, the azomethine double bond adopts an *E* conformation and the benzene rings form a dihedral angle of 77.70 (7)°. In the crystal, mol­ecules are linked by O—H⋯N and C—H⋯O hydrogen bonds and arranged in a zigzag fashion, forming infinite chains parallel to the *c* axis, resulting in a graph-set *R*_2_^2^(9) motif.

## Related literature

For the biological activity of Schiff bases, see: Khan *et al.* (2009 ▶); Gerdemann *et al.* (2002 ▶); Samadhiya & Halve (2001 ▶). For a related structure, see: Liang *et al.* (2009 ▶). For graph-set motifs, see: Bernstein *et al.* (1995 ▶).
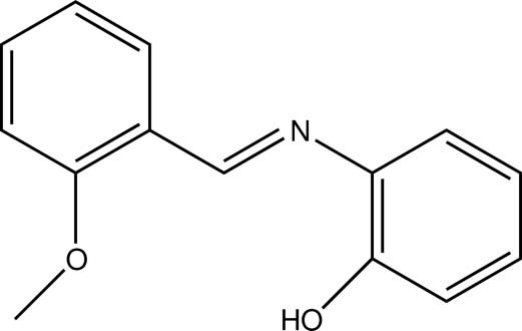


## Experimental

### 

#### Crystal data

C_14_H_13_NO_2_
*M* *_r_* = 227.25
Monoclinic, 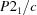

*a* = 9.8709 (5) Å
*b* = 6.6606 (3) Å
*c* = 18.6128 (9) Å
β = 105.249 (1)°
*V* = 1180.63 (10) Å^3^
*Z* = 4
Mo *K*α radiation
μ = 0.09 mm^−1^
*T* = 273 K
0.49 × 0.17 × 0.16 mm

#### Data collection

Bruker SMART APEX CCD area-detector diffractometer
Absorption correction: multi-scan (*SADABS*; Bruker, 2000 ▶) *T*_min_ = 0.959, *T*_max_ = 0.986
6660 measured reflections
2186 independent reflections
1680 reflections with *I* > 2σ(*I*)
*R*_int_ = 0.025

#### Refinement

*R*[*F* ^2^ > 2σ(*F* ^2^)] = 0.039
*wR*(*F* ^2^) = 0.100
*S* = 1.04
2186 reflections
159 parameters
H atoms treated by a mixture of independent and constrained refinement
Δρ_max_ = 0.16 e Å^−3^
Δρ_min_ = −0.14 e Å^−3^

### 

Data collection: *SMART* (Bruker, 2000 ▶); cell refinement: *SAINT* (Bruker, 2000 ▶); data reduction: *SAINT*; program(s) used to solve structure: *SHELXS97* (Sheldrick, 2008 ▶); program(s) used to refine structure: *SHELXL97* (Sheldrick, 2008 ▶); molecular graphics: *SHELXTL* (Sheldrick, 2008 ▶); software used to prepare material for publication: *SHELXTL*, *PARST* (Nardelli, 1995 ▶) and *PLATON* (Spek, 2009 ▶).

## Supplementary Material

Crystal structure: contains datablock(s) global, I. DOI: 10.1107/S1600536812016224/pv2528sup1.cif

Structure factors: contains datablock(s) I. DOI: 10.1107/S1600536812016224/pv2528Isup2.hkl

Supplementary material file. DOI: 10.1107/S1600536812016224/pv2528Isup3.cml

Additional supplementary materials:  crystallographic information; 3D view; checkCIF report

## Figures and Tables

**Table 1:** Hydrogen-bond geometry (Å, °)

*D*—H⋯*A*	*D*—H	H⋯*A*	*D*⋯*A*	*D*—H⋯*A*
O2—H2*A*⋯N1^i^	0.88 (2)	1.94 (2)	2.796 (2)	163 (2)
C10—H10*A*⋯O2^i^	0.93	2.56	3.269 (2)	133

Symmetry code: (i) 
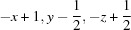
.

**Fig. 1.**
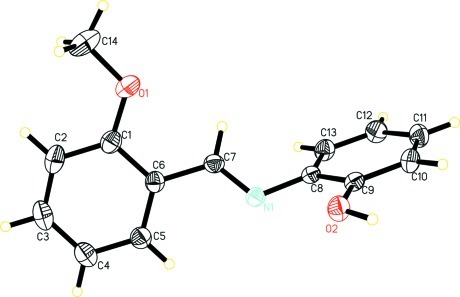
The molecular structure of the title compound with the atom numbering scheme. Displacement ellipsoids are drawn at the 30% probability level. H atoms are presented as small spheres of arbitrary radius.

**Fig. 2.**
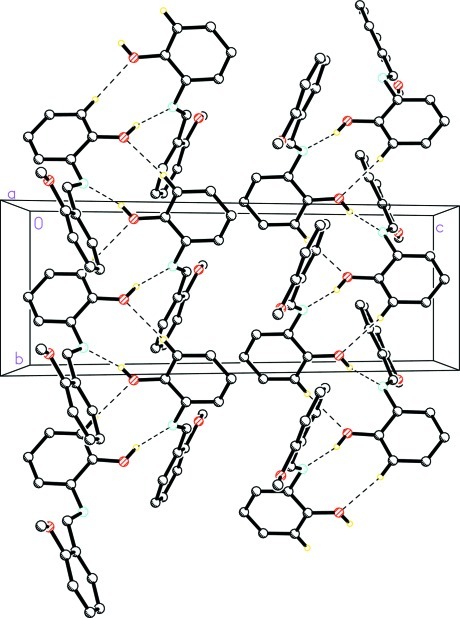
A view of the O—H···N and C—H···O hydrogen bonds (dotted lines) in the crystal structure of the title compound. H atoms non-participating in hydrogen-bonding were omitted for clarity.

## supplementary crystallographic information

### Comment

Schiff base ligands have remained an imperative part of research due to their used as intermediates and precursors for the synthesis of a variety of organic compounds having a broad range of biological activities (Khan *et al.*, 2009; Gerdemann *et al.*, 2002; Samadhiya & Halve, 2001). The title compound was prepared as a part of our ongoing research on bioactive compounds.

In the title molecule (Fig. 1), the azomethine (C═N, 1.2729 (18) Å) double bond adopts an E configuration. The bond lengths and angles in the title compound are similar to the corresponding bond lenghts and bond angles reported in a closely related compound, 2-(2,3,4-trimethoxy-6-methylbenzylideneamino)phenol (Liang *et al.*, 2009). The crystal structure is stabilized by O2—H2A···N1 and C1—H1B···O1 intermolecular hydrogen bonds resulting in chains of molecules lying parallel to the *c*-axis in a zig zag fashion (Fig. 2 and Tab. 1). The molecules lying about screw axis parallel to the *c*-axis form 9-membered rings due to hydrogen bonds in a motif with graph set R_2_^2^(9) (Bernstein *et al.*, 1995).

### Experimental

A mixture of 2-methoxybenzaldehyde (0.01 mol, 1.36 g) and 2-aminophenol (0.01 mol, 1.09 g) in ethanol (50 ml) along with 3–4 drops of conc. H_2_SO_4_ was refluxed for 3 h at 343 K. After cooling, the mixture was concentrated to one third of its volume under reduced pressure. The concentrated reaction mixture was kept at room temperature and light yellow crystals were obtained after five days. The crystalline product was collected, washed with methanol and dried to afford the title compound in 79% yield. Slow evaporation of a methanol solution afforded light yellow crystals suitable for single-crystal X-ray diffraction studies. All chemicals were purchased from Sigma-Aldrich.

### Refinement

The H atoms were positioned geometrically with C—H = 0.93 and 0.96 Å for aryl and methyl type H-atoms and constrained to ride on their parent atoms with *U*_iso_(H) = 1.2*U*_eq_(aryl-C) or 1.5*U*_eq_(methyl-C). The H atom on the oxygen was located from a difference Fourier map and refined isotropically. A rotating group model was applied to the methyl group.

### Crystal data

**Table:**

C_14_H_13_NO_2_	*F*(000) = 480
*M**_r_* = 227.25	*D*_x_ = 1.279 Mg m^−^^3^
Monoclinic, *P*2_1_/*c*	Mo *K*α radiation, λ = 0.71073 Å
Hall symbol: -P 2ybc	Cell parameters from 1522 reflections
*a* = 9.8709 (5) Å	θ = 2.7–24.5°
*b* = 6.6606 (3) Å	µ = 0.09 mm^−^^1^
*c* = 18.6128 (9) Å	*T* = 273 K
β = 105.249 (1)°	Block, colorless
*V* = 1180.63 (10) Å^3^	0.49 × 0.17 × 0.16 mm
*Z* = 4	

### Data collection

**Table:**

Bruker SMART APEX CCD area-detector diffractometer	2186 independent reflections
Radiation source: fine-focus sealed tube	1680 reflections with *I* > 2σ(*I*)
Graphite monochromator	*R*_int_ = 0.025
ω scan	θ_max_ = 25.5°, θ_min_ = 2.1°
Absorption correction: multi-scan (*SADABS*; Bruker, 2000)	*h* = −11→11
*T*_min_ = 0.959, *T*_max_ = 0.986	*k* = −8→8
6660 measured reflections	*l* = −22→22

### Refinement

**Table:**

Refinement on *F*^2^	Primary atom site location: structure-invariant direct methods
Least-squares matrix: full	Secondary atom site location: difference Fourier map
*R*[*F*^2^ > 2σ(*F*^2^)] = 0.039	Hydrogen site location: inferred from neighbouring sites
*wR*(*F*^2^) = 0.100	H atoms treated by a mixture of independent and constrained refinement
*S* = 1.04	*w* = 1/[σ^2^(*F*_o_^2^) + (0.048*P*)^2^ + 0.0868*P*] where *P* = (*F*_o_^2^ + 2*F*_c_^2^)/3
2186 reflections	(Δ/σ)_max_ < 0.001
159 parameters	Δρ_max_ = 0.16 e Å^−^^3^
0 restraints	Δρ_min_ = −0.14 e Å^−^^3^

### Special details

**Table:**

Geometry. All e.s.d.'s (except the e.s.d. in the dihedral angle between two l.s. planes) are estimated using the full covariance matrix. The cell e.s.d.'s are taken into account individually in the estimation of e.s.d.'s in distances, angles and torsion angles; correlations between e.s.d.'s in cell parameters are only used when they are defined by crystal symmetry. An approximate (isotropic) treatment of cell e.s.d.'s is used for estimating e.s.d.'s involving l.s. planes.
Refinement. Refinement of *F*^2^ against ALL reflections. The weighted *R*-factor *wR* and goodness of fit *S* are based on *F*^2^, conventional *R*-factors *R* are based on *F*, with *F* set to zero for negative *F*^2^. The threshold expression of *F*^2^ > σ(*F*^2^) is used only for calculating *R*-factors(gt) *etc*. and is not relevant to the choice of reflections for refinement. *R*-factors based on *F*^2^ are statistically about twice as large as those based on *F*, and *R*- factors based on ALL data will be even larger.

### Fractional atomic coordinates and isotropic or equivalent isotropic displacement parameters (Å^2^)

**Table:**

	*x*	*y*	*z*	*U*_iso_*/*U*_eq_	
O1	0.93139 (12)	0.87882 (18)	0.09546 (8)	0.0651 (4)	
O2	0.51633 (11)	0.57038 (17)	0.23910 (6)	0.0461 (3)	
H2A	0.479 (2)	0.490 (3)	0.2663 (11)	0.073 (6)*	
N1	0.57240 (12)	0.86217 (17)	0.15101 (6)	0.0371 (3)	
C1	0.90429 (16)	1.0512 (2)	0.12897 (9)	0.0452 (4)	
C2	0.99740 (17)	1.2103 (3)	0.14871 (9)	0.0564 (5)	
H2B	1.0849	1.2044	0.1389	0.068*	
C3	0.9600 (2)	1.3766 (3)	0.18280 (10)	0.0590 (5)	
H3A	1.0232	1.4824	0.1962	0.071*	
C4	0.83070 (19)	1.3892 (3)	0.19753 (9)	0.0546 (4)	
H4A	0.8062	1.5028	0.2203	0.066*	
C5	0.73841 (17)	1.2319 (2)	0.17814 (8)	0.0446 (4)	
H5A	0.6511	1.2399	0.1882	0.054*	
C6	0.77264 (15)	1.0608 (2)	0.14376 (8)	0.0384 (4)	
C7	0.67269 (15)	0.8955 (2)	0.12140 (8)	0.0381 (4)	
H7A	0.6830	0.8104	0.0836	0.046*	
C8	0.47548 (14)	0.7082 (2)	0.11917 (8)	0.0351 (3)	
C9	0.44428 (14)	0.5617 (2)	0.16613 (7)	0.0354 (3)	
C10	0.34863 (16)	0.4124 (2)	0.13671 (9)	0.0443 (4)	
H10A	0.3288	0.3132	0.1676	0.053*	
C11	0.28226 (16)	0.4101 (2)	0.06126 (9)	0.0482 (4)	
H11A	0.2182	0.3090	0.0418	0.058*	
C12	0.31018 (17)	0.5557 (2)	0.01490 (8)	0.0484 (4)	
H12A	0.2643	0.5546	−0.0356	0.058*	
C13	0.40674 (16)	0.7036 (2)	0.04392 (8)	0.0441 (4)	
H13A	0.4261	0.8018	0.0125	0.053*	
C14	1.0695 (2)	0.8464 (3)	0.08734 (13)	0.0799 (6)	
H14A	1.0744	0.7162	0.0661	0.120*	
H14B	1.1361	0.8538	0.1353	0.120*	
H14C	1.0910	0.9474	0.0552	0.120*	

### Atomic displacement parameters (Å^2^)

**Table:**

	*U*^11^	*U*^22^	*U*^33^	*U*^12^	*U*^13^	*U*^23^
O1	0.0531 (7)	0.0655 (8)	0.0875 (9)	0.0003 (6)	0.0376 (7)	−0.0084 (7)
O2	0.0529 (7)	0.0528 (7)	0.0330 (6)	−0.0114 (5)	0.0120 (5)	0.0027 (5)
N1	0.0413 (7)	0.0361 (7)	0.0367 (7)	−0.0026 (5)	0.0154 (6)	0.0003 (5)
C1	0.0434 (9)	0.0507 (10)	0.0437 (9)	−0.0014 (8)	0.0152 (7)	0.0060 (7)
C2	0.0411 (9)	0.0726 (12)	0.0549 (10)	−0.0129 (9)	0.0116 (8)	0.0120 (9)
C3	0.0628 (12)	0.0566 (11)	0.0502 (10)	−0.0233 (9)	0.0018 (9)	0.0037 (8)
C4	0.0654 (12)	0.0471 (10)	0.0487 (10)	−0.0101 (9)	0.0104 (9)	−0.0033 (8)
C5	0.0466 (9)	0.0450 (9)	0.0427 (9)	−0.0023 (8)	0.0126 (7)	0.0014 (7)
C6	0.0400 (8)	0.0394 (8)	0.0360 (8)	−0.0035 (7)	0.0100 (6)	0.0045 (6)
C7	0.0438 (9)	0.0369 (8)	0.0364 (8)	0.0000 (7)	0.0153 (7)	−0.0015 (6)
C8	0.0365 (8)	0.0357 (8)	0.0366 (8)	−0.0002 (6)	0.0159 (6)	−0.0022 (6)
C9	0.0345 (8)	0.0402 (8)	0.0337 (8)	0.0009 (6)	0.0129 (6)	0.0000 (6)
C10	0.0432 (9)	0.0440 (9)	0.0473 (9)	−0.0070 (7)	0.0145 (7)	0.0054 (7)
C11	0.0424 (9)	0.0505 (9)	0.0496 (9)	−0.0110 (7)	0.0084 (7)	−0.0063 (8)
C12	0.0485 (9)	0.0600 (10)	0.0346 (8)	−0.0048 (8)	0.0069 (7)	−0.0029 (7)
C13	0.0488 (9)	0.0484 (9)	0.0371 (8)	−0.0032 (8)	0.0149 (7)	0.0059 (7)
C14	0.0624 (12)	0.0986 (16)	0.0913 (15)	0.0195 (12)	0.0422 (11)	0.0109 (12)

### Geometric parameters (Å, º)

**Table:**

O1—C1	1.3665 (19)	C6—C7	1.464 (2)
O1—C14	1.427 (2)	C7—H7A	0.9300
O2—C9	1.3586 (16)	C8—C13	1.387 (2)
O2—H2A	0.88 (2)	C8—C9	1.3972 (19)
N1—C7	1.2729 (18)	C9—C10	1.382 (2)
N1—C8	1.4211 (18)	C10—C11	1.385 (2)
C1—C2	1.387 (2)	C10—H10A	0.9300
C1—C6	1.399 (2)	C11—C12	1.373 (2)
C2—C3	1.374 (3)	C11—H11A	0.9300
C2—H2B	0.9300	C12—C13	1.378 (2)
C3—C4	1.377 (3)	C12—H12A	0.9300
C3—H3A	0.9300	C13—H13A	0.9300
C4—C5	1.373 (2)	C14—H14A	0.9600
C4—H4A	0.9300	C14—H14B	0.9600
C5—C6	1.391 (2)	C14—H14C	0.9600
C5—H5A	0.9300		
			
C1—O1—C14	118.86 (15)	C13—C8—C9	119.10 (13)
C9—O2—H2A	111.2 (13)	C13—C8—N1	122.29 (12)
C7—N1—C8	117.39 (12)	C9—C8—N1	118.55 (12)
O1—C1—C2	124.55 (15)	O2—C9—C10	123.38 (13)
O1—C1—C6	115.58 (13)	O2—C9—C8	116.88 (12)
C2—C1—C6	119.88 (15)	C10—C9—C8	119.67 (13)
C3—C2—C1	119.88 (16)	C9—C10—C11	120.10 (14)
C3—C2—H2B	120.1	C9—C10—H10A	120.0
C1—C2—H2B	120.1	C11—C10—H10A	120.0
C2—C3—C4	121.09 (16)	C12—C11—C10	120.64 (14)
C2—C3—H3A	119.5	C12—C11—H11A	119.7
C4—C3—H3A	119.5	C10—C11—H11A	119.7
C5—C4—C3	119.15 (16)	C11—C12—C13	119.40 (14)
C5—C4—H4A	120.4	C11—C12—H12A	120.3
C3—C4—H4A	120.4	C13—C12—H12A	120.3
C4—C5—C6	121.41 (15)	C12—C13—C8	121.07 (14)
C4—C5—H5A	119.3	C12—C13—H13A	119.5
C6—C5—H5A	119.3	C8—C13—H13A	119.5
C5—C6—C1	118.58 (14)	O1—C14—H14A	109.5
C5—C6—C7	121.32 (13)	O1—C14—H14B	109.5
C1—C6—C7	120.08 (13)	H14A—C14—H14B	109.5
N1—C7—C6	123.41 (13)	O1—C14—H14C	109.5
N1—C7—H7A	118.3	H14A—C14—H14C	109.5
C6—C7—H7A	118.3	H14B—C14—H14C	109.5
			
C14—O1—C1—C2	7.6 (2)	C1—C6—C7—N1	158.03 (14)
C14—O1—C1—C6	−171.95 (16)	C7—N1—C8—C13	−53.20 (19)
O1—C1—C2—C3	−179.47 (14)	C7—N1—C8—C9	129.61 (14)
C6—C1—C2—C3	0.1 (2)	C13—C8—C9—O2	178.76 (13)
C1—C2—C3—C4	−0.4 (3)	N1—C8—C9—O2	−3.95 (19)
C2—C3—C4—C5	0.4 (2)	C13—C8—C9—C10	1.6 (2)
C3—C4—C5—C6	−0.2 (2)	N1—C8—C9—C10	178.92 (13)
C4—C5—C6—C1	−0.1 (2)	O2—C9—C10—C11	−178.05 (14)
C4—C5—C6—C7	−178.40 (14)	C8—C9—C10—C11	−1.1 (2)
O1—C1—C6—C5	179.72 (13)	C9—C10—C11—C12	−0.2 (2)
C2—C1—C6—C5	0.1 (2)	C10—C11—C12—C13	0.9 (2)
O1—C1—C6—C7	−1.9 (2)	C11—C12—C13—C8	−0.4 (2)
C2—C1—C6—C7	178.50 (14)	C9—C8—C13—C12	−0.9 (2)
C8—N1—C7—C6	174.21 (12)	N1—C8—C13—C12	−178.09 (14)
C5—C6—C7—N1	−23.6 (2)		

### Hydrogen-bond geometry (Å, º)

**Table:**

*D*—H···*A*	*D*—H	H···*A*	*D*···*A*	*D*—H···*A*
O2—H2*A*···N1^i^	0.88 (2)	1.94 (2)	2.796 (2)	163 (2)
C10—H10*A*···O2^i^	0.93	2.56	3.269 (2)	133

Symmetry code: (i) −*x*+1, *y*−1/2, −*z*+1/2.
